# Mammographic density and survival in interval breast cancers

**DOI:** 10.1186/bcr3440

**Published:** 2013-06-20

**Authors:** Louise Eriksson, Kamila Czene, Lena U Rosenberg, Sven Törnberg, Keith Humphreys, Per Hall

**Affiliations:** 1 Institution of Medical Epidemiology and Biostatistics, Karolinska Institutet, Nobels Väg 12A, S-171 77 Stockholm, Sweden; 2 Department of Clinical Sciences, Karolinska Institutet, Danderyd Hospital, Mörbygårdsvägen 5, S-182 88 Stockholm, Sweden; 3 Cancer Screening Unit, Oncologic Center, Norrbacka S3:00, Karolinska University Hospital, Solna, S-171 76 Stockholm, Sweden

## Abstract

**Introduction:**

Mammographic density (MD) is the strongest risk factor for breast cancer. It is also strongly associated with interval cancers (ICs) due to decreased screening sensitivity and possibly by also giving rise to more aggressive tumors. With this information as background, we compared survival in interval and screen-detected cancers, taking MD into consideration.

**Methods:**

The patients were postmenopausal women ages 50 to 74 years who were diagnosed with breast cancer in Sweden between 1993 and 1995. A total of 1,115 women with screen-detected cancers and 285 with ICs had available mammograms. Cox proportional hazards models were used to compare breast cancer-specific survival between interval and screen-detected cancers stratified on MD.

**Results:**

Hazard rates for breast cancer-specific survival were approximately three times higher in ICs than in screen-detected cancers, independent of MD. After adjustment for tumor size, a proxy for time to diagnosis, ICs in nondense breasts still had a statistically significantly increased hazard rate compared to screen-detected cancers in nondense breasts (5-yr survival hazard ratio (HR) 2.43, *P *= 0.001). In dense breasts, however, there was no longer evidence of a difference in survival between ICs and screen-detected cancers (5-yr survival HR 1.41, *P *= 0.486).

**Conclusions:**

In nondense breasts, ICs seem to be truly more aggressive than screen-detected cancers. In dense breasts, the poorer prognosis of ICs compared to that of screen-detected cancers may be attributable at least partially to later detection. However, to the best of our knowledge, this study is the first to investigate these relationships, and further studies are warranted to confirm our results.

## Introduction

Mammographic density (MD) is one of the most important risk factors for breast cancer, with women with the highest breast density (above 75%) having a four- to sixfold increased risk compared with women with completely fatty breasts [1]. MD is determined by the tissue composition of the breast. The epithelium and fibrous tissue are radiodense and appear white on a mammogram, whereas the fatty tissue is radiolucent and appears black. MD is often given as a percentage (percentage density (PD)), which is calculated by dividing the dense area by the total breast area. Consequently, larger amounts of fibroglandular tissue in relation to fatty tissue will lead to higher PD and vice versa.

Interval cancers (ICs) are cancers diagnosed after a negative screening and before the next planned screening mammography. ICs are usually separated into "true" ICs, that is, cancers that could not be detected on the previous mammogram (and thus truly have arisen within the screening interval) and cancers missed at a previous mammography, that is, false-negative examinations. The former are thought to be more aggressive and have a poorer prognosis than screen-detected (SD) cancers [2]. Because MD and tumors both appear white on a mammogram, extensive density can hide tumors, a phenomenon referred to as *masking*. Density thus decreases mammographic sensitivity [3], increasing the risk of IC [4]. Whether MD also gives rise to more highly proliferative tumors is unknown. This is theoretically plausible because MD has consistently been shown to be associated with the stromal composition of the breast [5-9], and the stroma plays an important part in tumor progression [10]. There is also some evidence that MD is associated with more aggressive tumor characteristics [11-13], but most studies have found no association between MD and tumor characteristics [14-19].

Boyd *et al*. studied MD and breast cancer risk according to mode of detection [4]. They found that women with PD 75% or higher had an odds ratio of 17.8 for IC within 12 months after a negative screening, but that the increase in risk did not persist after these 12 months [4]. The authors interpreted this difference to be in support of masking, rather than rapid growth, causing the association between MD and ICs. However, it has previously been proposed that tumors with higher growth rates are overrepresented among tumors detected early in the interval between two screening mammographies [20].

In this study, we aimed to investigate breast cancer-specific survival comparing ICs to SD cancers, taking MD into consideration. To the best of our knowledge, this has never previously been studied. We postulated that ICs arising in nondense breasts, that is, breasts in which mammographic screening sensitivity is high, are to a greater extent "true" ICs than ICs arising in dense breasts, which, to a greater extent, are due to false-negative examinations as a consequence of masking. We further hypothesized that this would be reflected in shorter survival for ICs detected in nondense breasts than in dense breasts, at least after adjusting for tumor size, a proxy for tumor age.

## Materials and methods

### Study population

This study is an extension of a large case-control study among all Swedish residents born in Sweden and ages 50 to 74 years at the time of enrollment between 1 October 1993 and 31 March 1995. Details regarding data collection and subjects have been described previously [21]. Women with incident primary invasive breast cancer were identified via the six Swedish Regional Cancer Registries. The study identified 3,979 women with breast cancer, of whom 84% (*n *= 3,345) participated. However, 19 were diagnosed outside the study period, 1 had a diagnosis other than breast cancer and 58 had noninvasive breast cancer, rendering them ineligible and leaving 3,267 women in the study.

For this study, the inclusion criteria were further refined to include only postmenopausal women who had no prior diagnosis of cancer other than nonmelanoma skin cancer (see Eriksson *et al. *[14] for details). The study base thus consisted of 2,720 breast cancer cases.

Details on mammography retrieval have been described elsewhere [14]. For the eligible participants in this study, we managed to collect mammograms of 2,046 women (75%). However, 107 women who had only postdiagnostic mammograms were excluded, as were 65 women lacking mammograms of the breast contralateral to the tumor and 79 women lacking postmenopausal mammograms. The latter exclusion is due to the fact that MD may differ histologically in pre- and postmenopausal women [8] and also may be affected differentially by hormones [22,23]. Images of poor quality were omitted, which led to the exclusion of 21 women. The median difference from date of mammography to study entrance was 50 days.

Descriptive characteristics of the women excluded due to the above-described factors did not differ from women who were included in the study (*n *= 1,774). These characteristics were body mass index (BMI), breast cancer heredity, previous benign breast disease, age at menarche, oral contraceptive use, age at first birth, parity, breastfeeding, age at menopause and hormone replacement therapy (HRT). However, there was a difference in mean age between the excluded and included women (62.9 years for included women compared to 63.6 years for excluded women, *P *= 0.015).

Approval of the study was given by the ethical review boards in the respective regions in which the subjects were based, that is, the Regional Ethical Review Board in Gothenburg, the Regional Ethical Review Board in Linköping, the Regional Ethical Review Board in Malmö-Lund, the Regional Ethical Review Board in Stockholm (Karolinska Institutet), the Regional Ethical Review Board in Umeå and the Regional Ethical Review Board in Uppsala. Prior to participation via a mailed questionnaire, written consent was obtained from all patients.

### Data collection and classification

Data on sociodemographic, anthropometric, hormonal and lifestyle factors were collected by means of a mailed questionnaire. Because the date of mammography was prior to study entrance, the variables age, menopausal status and HRT were reassessed according to the date of mammography. This was not possible for BMI, as we had information on BMI only at study entrance and 1 yr prior to study entrance. However, it has previously been shown that interindividual variations in BMI are small [24] and the difference in BMI at study entrance and 1 yr prior to this was 0.05 U (standard deviation 1.2 U) for our study participants.

HRT was classified according to recency (current, former or never). Because the influence of HRT on MD diminishes within 3 wk of cessation [25], former users were those who had discontinued HRT more than 1 mo prior to the date of mammography. All compounds, modes of administration and potencies were included in the HRT variable, except for low-potency, estrogen-only pharmaceuticals, because the latter have not been shown to increase breast cancer risk [21].

We collected information on tumor characteristics and reason for diagnostic mammography from surgical and oncological patient records throughout Sweden. We also visited 66 of the 68 units performing mammographic examinations in Sweden and collected information on the dates and reasons for the examinations (screening or referral and reason for referral) performed within 5 yr before diagnosis, excluding 3 mo just before diagnosis to avoid registering diagnostic examinations. To evaluate whether lack of information on prediagnostic mammographies was due to failure to find the information or a true lack of previous mammographies, we compared the information retrieved from mammography units to the questionnaire data. In the latter, women had stated how many mammographies they had undergone 5 yr prior to diagnosis.

Grade was classified into three groups according to the Nottingham histologic grade or the Bloom-Richardson scale [26]. Tumors were considered estrogen receptor (ER)-positive or progesterone receptor (PR)-positive if they contained at least 0.05 fmol receptor/μg DNA or at least 10 fmol receptor/mg protein. Cells in S-phase, a marker of proliferation, were given as a percentage by each laboratory. However, this value was then dichotomized based on the threshold value for high S-phase fraction calculated at each laboratory. For one laboratory, we lacked this information and instead took the mean value for all laboratories as the threshold.

We collected information on emigrations from the Swedish National Population Registry and the date and cause of death until 31 December 2008 from the Swedish Causes of Death Registry. The latter registry covers all residents in Sweden with essentially no missing deaths and has been shown to correctly classify 98% of breast cancer deaths [27]. The follow-up is thus virtually complete.

### Assessment of mode of detection

The following definition was applied to assess IC status: non-SD cancers (that is, reason for diagnostic mammography was not screening) where a previous, negative (either negative directly at mammography or at follow-up) screening mammography had been conducted 3 to 24 mo before the diagnostic mammography. Twenty-four months was the screening interval in Sweden during the study. The 3-mo cut-off was used to avoid including mammography screenings with clinical workup periods. Of the 1,774 women, 1,115 had SD cancers and 285 had ICs. A total of 268 women had non-SD cancers but either a previous screening mammography more than 2 yr prior to diagnosis or no previous screening mammography at all. These patients were excluded from further analyses. A total of 106 women lacked information to be able to assess IC status and were also excluded. Our final study population thus consisted of 1,115 women with SD cancers and 285 women with ICs.

### Mammographic density data

Film mammograms of the mediolateral oblique view were digitized using an Array 2905 HD Laser Film Digitizer (Array Corp, Tokyo, Japan), which covers a range of 0 to 4.7 optical density. The mediolateral oblique view was used because this was the routine view used at mammography screening in Sweden. The density resolution was set at 12-bit spatial resolution. We used Cumulus, a computer-assisted thresholding technique, to assess density [28] of the mammogram contralateral to the tumor. For each image, a trained observer (LE) set the appropriate grayscale threshold levels defining the edge of the breast and distinguishing dense from nondense tissue. The software was used to calculate the total number of pixels within the entire region of interest and within the region identified as dense. PD was then calculated using these values (dense area divided by total breast area). The images were measured together with approximately the same amount of images for healthy women, and the reader was blinded to case-control status, IC status and survival. A random 10% of the images were included as replicates to assess the intraobserver reliability, which was high at an *R*^2 ^value of 0.92.

#### Statistical analyses

Because we included only postmenopausal women and their mean age was 62 years, the density distribution was relatively low and more homogeneous than it would be in a younger and/or combined pre- and postmenopausal population. To compare dense versus nondense breasts, we used an *a priori *defined cut-off of mammographic density at 25%, defining the highest quartile in our cohort, that is, 25% of the study population had a PD of at least 25%. A benefit of this cut-off is that it also differentiates fatty (nondense) from dense breasts according to the Breast Imaging Reporting and Data System (BI-RADS) (BI-RADS category 1 correlates to a PD <25%) [29]. However, it is important to note that visual estimation of density using BI-RADS may overestimate PD compared to computer-assisted quantitative methods [30], so these two methods may not be directly comparable.

Patient characteristics were compared among women with SD cancers and ICs using χ^2 ^tests of association and Student's *t*-test. Tumor characteristics were compared on the basis of mode of detection (SD vs IC, then also stratifying on density), using χ^2 ^tests of association and Student's *t*-test.

The Cox proportional hazards model was used to calculate hazard ratios (HRs) and their associated 95% confidence intervals (95% CIs) for 5-yr breast cancer-specific survival. Age at mammography (continuous), BMI (continuous) and HRT (categorical) were included as possible confounders in the first model, also adjusting for tumor size (continuous) in our second model and further adjusting for lymph node metastasis (categorical (zero, one to three or four or more affected lymph nodes)) in our third model. The second model was viewed as our main model. We carried out overall analyses as well as analyses stratified by density. To examine differences in HRs (comparing interval and SD cancers) between density groups, we tested for interaction between density and mode of detection (SD vs IC) in analyses including all women. All analyses were carried out using STATA version 12.1 statistical software (StataCorp, College Station, TX, USA).

## Results

Compared with women with SD cancers, women with ICs were younger (60.9 years of age vs 62.0, *P *= 0.012), had lower BMI (25.2 vs 26.1, *P *< 0.001), higher PD (*P *< 0.001), were more often (past) users of oral contraceptives (*P *= 0.003), and were more often ever-users of HRT (*P *< 0.001) (Table 1).

**Table 1:** Descriptive statistics comparing screen-detected cancers and interval cancers

Characteristics	Screen-detected cancers (*n *= 1,115)	Interval cancers (*n *= 285)	*P *value
Mean age at mammography (yr)	62.0	60.9	0.012

Mean BMI	26.1	25.2	<0.001

Mean age at menarche (yr)	13.5	13.5	0.442

Oral contraceptive use, *n *(%)			0.003

Yes	358 (32%)	118 (42%)	

No	754 (68%)	165 (58%)	

Mean age at first birth (yr)	25.4	25.0	0.172

Mean parity (*n*)	1.8	1.9	0.775

Breastfeeding, *n *(%)			0.152

Yes	777 (78%)	211 (82%)	

No	219 (22%)	46 (18%)	

Mean age at menopause (yr)	50.4	50.2	0.302

HRT use, *n *(%)			<0.001

Never	800 (73%)	160 (57%)	

Past	247 (22%)	71 (25%)	

Current	54 (5%)	52 (18%)	

Previous benign breast disease, *n *(%)			0.182

Yes	146 (13%)	46 (16%)	

No	965 (87%)	238 (84%)	

Breast cancer heredity^a^, *n *(%)			0.434

Yes	160 (15%)	46 (17%)	

No	923 (85%)	230 (83%)	

Percentage density (PD), *n *(%)			<0.001

<5%	240 (22%)	38 (13%)	

5% to <10%	207 (19%)	50 (18%)	

10% to <25%	420 (38%)	100 (35%)	

25% to <50%	214 (19%)	79 (28%)	

≥50%	34 (3%)	18 (6%)	

^a^Family history of breast cancer in a first-degree relative. BMI, body mass index; HRT, hormone replacement therapy.

There was a varying degree of missing data for tumor characteristics: About 1% lacked information on tumor size and histopathological classification; approximately 30% lacked information on grade, ER status and PR status; and 61% lacked information on proliferation rate. We chose to include the latter group in our comparison of patient characteristics across women with SD cancers and ICs only for exploratory analysis. The results on proliferation rate should be interpreted with extreme caution. Human epidermal growth factor receptor 2 (HER2) status was not assessed, because women were diagnosed between 1993 and 1995.

ICs had a particularly unfavorable phenotype compared to SD cancers. They were larger (*P *< 0.001), more often lymph node-positive (*P *< 0.001), ER-negative (*P *= 0.020), PR-negative (*P *= 0.008), of higher grade (*P *< 0.001) and had a higher proliferation rate (*P *< 0.001) (Table 2). The differences in tumor characteristics between ICs and SD cancers were largely unchanged when only nondense breasts or only dense breasts were considered. In dense breasts, however, only differences in tumor size (*P *< 0.001), lymph node metastasis (*P *= 0.001) and grade (*P *= 0.012) were statistically significant. Furthermore, despite the difference in tumor size, there was no statistically significant difference in proliferation rates (*P *= 0.523) between ICs and SD cancers in dense breasts.

**Table 2:** Descriptive statistics of distributions of tumor characteristics comparing interval cancers to screen-detected cancers, interval cancers to screen-detected cancers limited to nondense breasts and interval cancers to screen-detected cancers limited to dense breasts

	All (*n *= 1,400)			Nondense, PD <25% (*n *= 1,055)			Dense, PD ≥25% (*n *= 345)		
	**SD cancer** **(*n *= 1,115)**	**IC** **(*n *= 285)**	***P *value**	**SD cancer** **(*n *= 867)**	**IC** **(*n *= 188)**	***P *value**	**SD cancer** **(*n *= 248)**	**IC** **(*n *= 97)**	***P *value**

Mean tumor size (mm)	14.4	20.5	<0.001	14.2	19.6	<0.001	15.2	22.4	<0.001

Lymph node metastasis, *n *(%)			<0.001			<0.001			0.001

Positive	240 (22%)	109 (39%)		190 (22%)	72 (39%)		50 (20%)	37 (38%)	

Negative	856 (78%)	171 (61%)		660 (78%)	111 (61%)		196 (80%)	60 (62%)	

ER status, *n *(%)			0.020			0.054			0.150

Positive	627 (83%)	162 (76%)		492 (82%)	99 (75%)		135 (84%)	63 (77%)	

Negative	131 (17%)	52 (24%)		106 (18%)	33 (25%)		25 (16%)	19 (23%)	

PR status, *n *(%)			0.008			0.018			0.133

Positive	536 (72%)	130 (62%)		420 (71%)	78 (60%)		116 (74%)	52 (65%)	

Negative	211 (28%)	79 (38%)		171 (29%)	51 (40%)		40 (26%)	28 (35%)	

Grade, *n *(%)			<0.001			0.008			0.012

1	147 (20%)	20 (11%)		116 (20%)	15 (14%)		31 (19%)	5 (6%)	

2	333 (45%)	71 (37%)		265 (45%)	40 (36%)		68 (42%)	31 (39%)	

3	268 (36%)	99 (52%)		205 (35%)	56 (50%)		63 (39%)	43 (54%)	

Histological classification, *n *(%)			0.703			0.872			0.441

Ductal	813 (73%)	206 (74%)		640 (74%)	134 (72%)		173 (71%)	72 (76%)	

Lobular	125 (11%)	35 (13%)		92 (11%)	22 (12%)		33 (13%)	13 (14%)	

Other	173 (16%)	39 (14%)		134 (15%)	29 (16%)		39 (16%)	10 (11%)	

Proliferation rate			<0.001			<0.001			0.523

High	108 (25%)	52 (42%)		83 (25%)	36 (48%)		25 (28%)	16 (33%)	

Low	316 (75%)	71 (58%)		252 (75%)	39 (52%)		64 (72%)	32 (67%)	

^a^IC, interval cancer; ER, estrogen receptor; PD, percentage density; PR, progesterone receptor; SD, screen-detected.

According to the Cox proportional hazards model, ICs had a worse prognosis than SD cancers, independent of adjustments (Table 3). Both types of ICs were associated with a HR of about 3 before adjustment for tumor size. After adjustment for tumor size, ICs in nondense breasts still had a statistically significant worse survival than SD cancers in nondense breasts (HR 2.43, 95% CI 1.44 to 4.10). In dense breasts, the point estimate approached unity and was statistically nonsignificant (HR 1.41, 95% CI 0.53 to 3.74). The difference between these two HRs was not statistically significant (*P *= 0.304). When we additionally adjusted for lymph node metastases, point estimates for 5-yr breast cancer-specific survival were further attenuated for all comparison groups. In nondense breasts, however ICs still had a statistically significant worse survival than SD cancers (HR 1.76, 95% CI 1.01 to 3.09).

**Table 3:** Hazard ratios comparing 5-year breast cancer-specific survival of women with interval cancers with women with screen-detected cancers, only including nondense breasts versus only including dense breasts

	All			Nondense breasts			Dense breasts			***P *value for interaction term**^a^
	
	HR	95% CI	*P *value	HR	95% CI	*P *value	HR	95% CI	*P *value	
Model 1^b^	3.50	2.25 to 5.44	<0.001	3.62	2.17 to 6.06	<0.001	3.00	1.26 to 7.17	0.013	0.742

Main model (model 2)^c^	2.17	1.36 to 3.47	0.001	2.43	1.44 to 4.10	0.001	1.41	0.53 to 3.74	0.486	0.304

Model 3^d^	1.69	1.03 to 2.76	0.038	1.76	1.01 to 3.09	0.047	1.26	0.47 to 3.38	0.649	0.337

^a^Test of interaction between density and mode of detection (screen-detected cancer vs interval cancer). CI, confidence interval; HR, hazard ratio.

^b^Adjusted for age, body mass index (BMI) and hormone replacement therapy (HRT) use.

^c^Adjusted for age, BMI, HRT use and tumor size.

^d^Adjusted for age, BMI, HRT use, tumor size and lymph node metastasis.

## Discussion

In this study, we have shown that women with ICs in nondense and dense breasts (defined as PD less than 25% and PD at least 25%, respectively) had poorer survival than those with corresponding SD cancers before adjusting for tumor size, a proxy for time to diagnosis. After adjusting for tumor size, ICs in nondense breasts still had a HR greater than 2 compared to SD cancers in nondense breasts. The former thus seem to be truly more aggressive tumors. For women with dense breasts, there was no longer evidence of a statistically significant difference between ICs and SD cancers after adjusting for tumor size. Hence, the poorer prognosis of ICs in dense breasts compared to corresponding SD cancers may be due to differences in time to diagnosis, but our results need confirmation in independent and larger data sets.

We adjusted for tumor size to account for differences in lead time. This is not entirely unproblematic, because it may under- or overestimate the effect of lead time. On the one hand, tumor size is a product of both time and proliferation rate and is therefore indirectly associated with tumor aggressiveness. Hence, adjustment for tumor size may also reduce differences in aggressiveness. On the other hand, one could argue that adjustment for tumor size alone may not fully account for differences in time to diagnosis; instead, adjustment for both tumor size and lymph node metastasis should be carried out [31]. Although lymph node metastasis also is a marker of chronology, however, presence of lymph node metastasis has been shown to reflect a more aggressive phenotype, directly affecting survival [32]. Adjustment for lymph node metastasis will therefore risk depleting differences in aggressiveness, which is the factor we wished to study. When we additionally adjusted for lymph node metastasis, point estimates for 5-yr breast cancer-specific survival were further attenuated for all comparison groups. Notably, in nondense breasts, women with ICs still had a statistically, significant poorer survival than those with SD cancers.

We defined ICs as cancers developing 3 to 24 mo after a negative screening mammography. The 3-mo cut-off was set to avoid including the clinical workup period sometimes required for diagnosis. Hence, some ICs may have been excluded from this study. According to our definition, about 20% (285/(285 + 1,115)) of the total number of cancers in screened women were ICs. This is slightly lower than previously published results for ICs in screened women in Stockholm, Sweden, diagnosed between 1989 and 1992, in which the proportion was approximately 26% [33]. However, that study also included premenopausal women, which would increase the proportion of women with ICs somewhat because they have denser breasts [34]. Thus, the cut-off at 3 mo does not seem to have decreased the number of ICs markedly.

Our study has several strengths: the population-based study design; study size; detailed covariate information; and quantitative, semiautomated density measurements to minimize exposure misclassification [35]. A limitation of our study is that our study population was restricted to postmenopausal women. Our results may not be applicable to premenopausal populations; hence, studies are needed to investigate the relationship between mammographic density, ICs and survival in premenopausal women.

## Conclusions

Women diagnosed with ICs in nondense breasts seemed to have a particularly aggressive phenotype and worse prognosis compared to women with SD cancers in nondense breasts. If this novel finding holds true, a future aim should be to establish the risk factors associated with the more aggressive entity of ICs in nondense breasts. Further research on mechanisms that trigger such cancers may inform treatment strategies and, ultimately, our approaches to prevention. In women with dense breasts, we found some support for the hypothesis that the poorer prognosis seen in women diagnosed with ICs is due to later detection. If confirmed by other studies, this observation emphasizes the need for screening techniques with higher sensitivity than conventional mammography in women with dense breasts.

## Abbreviations

BMI: body mass index; CI: confidence interval; ER: estrogen receptor status; HER2: human epidermal growth factor receptor 2; HR: hazard ratio; HRT: hormone replacement therapy; IC: interval cancer; MD: mammographic density; PD: percentage density; PR: progesterone receptor; SD: screen-detected cancer

## Competing interests

The authors declare that they have no competing interests.

## Authors' contributions

LE and LR participated in the collection of the data. LE carried out the density measurements. LE carried out the statistical analyses with help from KH. All authors participated in the study design and interpretation of results. LE wrote the first draft of the manuscript. All authors participated in the revision of the manuscript and read and approved the final manuscript.
